# High-efficiency expression and secretion of human FGF21 in *Bacillus subtilis* by intercalation of a mini-cistron cassette and combinatorial optimization of cell regulatory components

**DOI:** 10.1186/s12934-019-1066-4

**Published:** 2019-01-28

**Authors:** Dandan Li, Gang Fu, Ran Tu, Zhaoxia Jin, Dawei Zhang

**Affiliations:** 1 School of Biological Engineering, Dalian Polytechnic University, Dalian, 116034 People’s Republic of China; 2 Tianjin Institute of Industrial Biotechnology, Chinese Academy of Sciences, Tianjin, 300308 People’s Republic of China; 3 Key Laboratory of Systems Microbial Biotechnology, Chinese Academy of Sciences, Tianjin, 300308 People’s Republic of China

**Keywords:** FGF21, *Bacillus subtilis*, Heterologous protein expression, Mini-cistron, Chaperone, Signal peptide

## Abstract

**Background:**

Recombinant human Fibroblast growth factor 21 (rhFGF21) is an endocrine hormone that has profound effects on treatment of metabolic diseases. However, rhFGF21 is prone to form inclusion body when expressed in bacteria, which results in, the downstream process of purification of bioactive rhFGF21 is time-consuming and labor intensive. The aim of this work is to explore a new method for improving the soluble expression and secretion level of rhFGF21 in *B. subtilis*.

**Results:**

A codon optimized rhFGF21 gene was expressed under the control of a strong inducible promoter P_*malA*_ in *B. subtilis*. A mini-cistron cassette (from *gsiB*) was located upstream of rhFGF21 in expression vector (pMATEFc5), which could reduce the locally stabilized mRNA secondary structure of transcripts and enhance the efficiency of translation initiation. Then various chaperones were further overexpressed to improve the expression efficiency of rhFGF21. Results showed that overexpression of the chaperone DnaK contributed to the increase of solubility of rhFGF21. Moreover, an extracellular proteases deficient strain *B. subtilis* Kno6cf was used to accumulate the secreted rhFGF21 solidly. In addition, eleven signal peptides from *B. subtilis* were evaluated and the SP_*dacB*_ appeared the highest secretion yield of rhFGF21 in *B. subtilis*. Finally, the combinatorial optimized strain achieved an about ninefold increase of the soluble rhFGF21 production after 24 h of flask fermentation in comparison with the initial production strain.

**Conclusion:**

This work provided a comprehensive strategy for secretory expressing the heterologous protein rhFGF21 in *B. subtilis.* To our knowledge, this is the first report of the highly efficient production of rhFGF21 in *B. subtilis* and this approach may provide some suggestions for heterologous proteins production in *B. subtilis*.

**Electronic supplementary material:**

The online version of this article (10.1186/s12934-019-1066-4) contains supplementary material, which is available to authorized users.

## Background

Human fibroblast growth factor 21 (hFGF21) belongs to the fibroblast growth factor (FGF) superfamily which is widely expressed in fetal and adult tissues and directs cell growth and cell differentiation [1]. The full-length hFGF21 consists of 209 amino acids with a signal peptide of 28 amino acids at the N-terminus, splicing a mature FGF21 polypeptide of 181 amino acids. Besides, a disulfide bond (Cys75-Cys93) was observed in the mature FGF21 polypeptide, which is buried in the internal core of the FGF21 core domain, and seems hard to be attacked by reducing reagent [2]. As a novel endocrine hormone, FGF21 has profound effects on the regulation of metabolic parameters such as glucose and lipid homeostasis, and represents a promising potential therapeutic target in type 2 diabetes (T2D) and obesity [3]. For example, animal studies demonstrated that adapting FGF21 in obese animals could lead to improvements in insulin sensitivity, weight loss, decrease in low-density lipoprotein cholesterol levels, and reversal of hepatic steatosis [4]. With the increase in metabolic syndrome patients [5], FGF21 could potentially be a revolutionary new way to treat obesity, T2D and other metabolic diseases. In particular, recombinant human fibroblast growth factor 21 (rhFGF21) has been a focus of investigation as a new drug candidate for metabolic diseases [3].

Thus, to increase the production of recombinant human fibroblast growth factor 21 protein and variants for academic and medical research, rhFGF21 was expressed using heterologous hosts such as *Pichia pastoris* (*P. pastoris*) [6] and *Escherichia coli* (*E. coli*) [7, 8]. However, *P. pastoris* as host cell grows much slower than bacterial cells, and needs lower temperature for cultivation [9–11], while intracellular expression of target proteins in *E. coli* brings about the increase of purification cost due to the cell disruption and removal of endotoxin [12]. In a previous study, Wang et al. [7] fused FGF21 with a small ubiquitin-related modifier (SUMO) to assist the protein folding process, and expressed the fused gene in *E*. *coli* BL21 (DE3). However, this strategy is unsuitable for industrial-scale production due to the high cost of SUMO protease and low protein recovery. In another attempt to increase the soluble expression level of rhFGF21, an artificial gene encoding the FGF21 sequence was constructed into a pET-3c vector and expressed in *E. coli* Origami BL21 (DE3) host cell which is suitable for protein post-translational folding process, but the final protein production is still low [8]. Compared with *E. coli*, *Bacillus subtilis* is a generally recognized as safe (GRAS) organism due to the lack of pathogenicity and deficiency in endotoxins [13]. *B. subtilis* has naturally high secretory capacity and exports proteins directly into the extracellular medium via the Sec or Tat secretion machinery [14, 15]. Several heterologous proteins such as β-mannanases, α-amylases and RDPE have been engineered to achieve high-level secretory expression in *B. subtilis* [16–18]. Thus, in consideration of cultivation condition, cell growth rate and secretory capacity, it seems that *B. subtilis* is a better candidate for large-scale protein secretory production. However, eukaryotic proteins expressed in *B. subtilis* often failed or showed non-functional due to the low level of transcription, translation or folding efficiency [19–21]. So, in order to achieve successfully secretory expression of eukaryotic proteins in *B. subtilis*, protein expression at the levels of transcription, translation, folding, translocation and transport should be optimized comprehensively.

In this study, we successfully expressed rhFGF21 in *B. subtilis* and subsequently designed a comprehensive expression strategy to increase the solubility and secretion efficiency of rhFGF21 through optimization at the levels of transcription, translation, protein folding, resistance to proteolysis, signal peptide optimization, and translocation efficiency in *B. subtilis* [22]. The rhFGF21 gene was expressed using a maltose-inducible promoter P_*malA*_ in *B. subtilis* 1A751 without any fusion partner to aid in expression and purification [23]. Next, to improve the translational efficiency of rhFGF21, seven additional mini-cistrons intercalated in the expression vector of rhFGF21 were constructed and evaluated. Furthermore, the production of soluble protein was further elevated by overexpression of different chaperones. At last, the rhFGF21 protein leading by the optimized signal peptide SP_*dacB*_ was successfully secreted into medium in a extracellular proteases deficient host strain Kno6. Comparing to other attempts of expression human originated protein such as rhGH [24] in *B. subtilis*, we are not just optimized expression elements such as promoter and signal peptide [25], but improved the efficiency of transcription, translation, protein folding, protease-resistance, translocation and transport by a comprehensive strategy in *B. subtilis*. To the best of our knowledge, this is the first report of the highly efficient production of rhFGF21 in *B. subtilis.*

## Results

### Strategies designed for improving rhFGF21 protein expression in *B. subtilis*

**Fig. 1:**
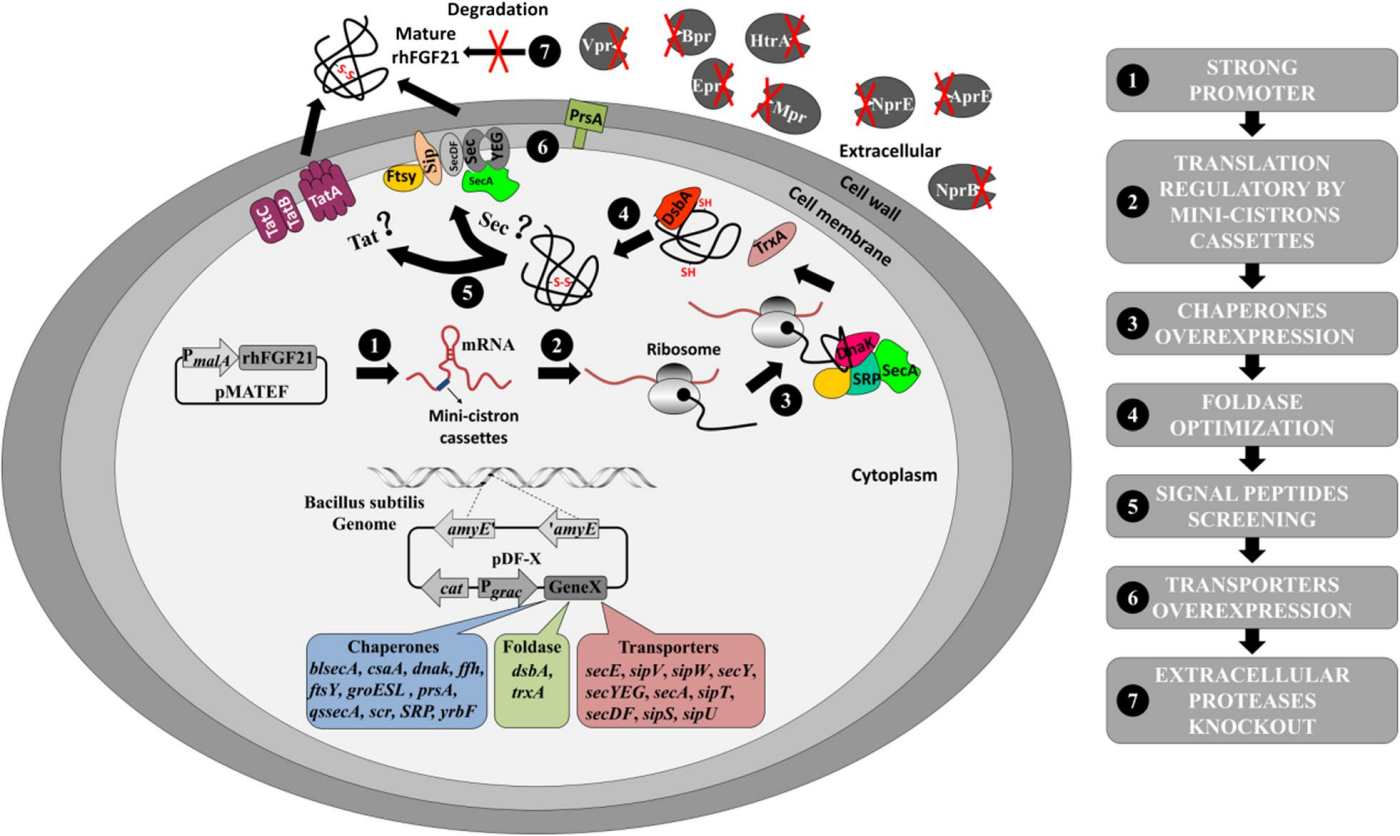
The flow diagram of experimental strategies in this study

In order to secretory express the target protein rhFGF21 in *B. subtilis*, different strategies at the level of transcription, translation, protein folding/degrading, translocation and secretion process were performed to address potential bottlenecks in eukaryotic protein expression (Fig. 1), which includes selection of strong promoters, introduction of a mini-cistron cassette to enhance translational initiation, overexpression of different chaperones, and selection of a optimum signal peptide. These strategies were combined to improve solubility, production and secretory efficiency of rhFGF21 in *B. subtilis*.

### Enhancing transcriptional strength of rhFGF21 in *B. subtilis* by using different promoters

**Fig. 2:**
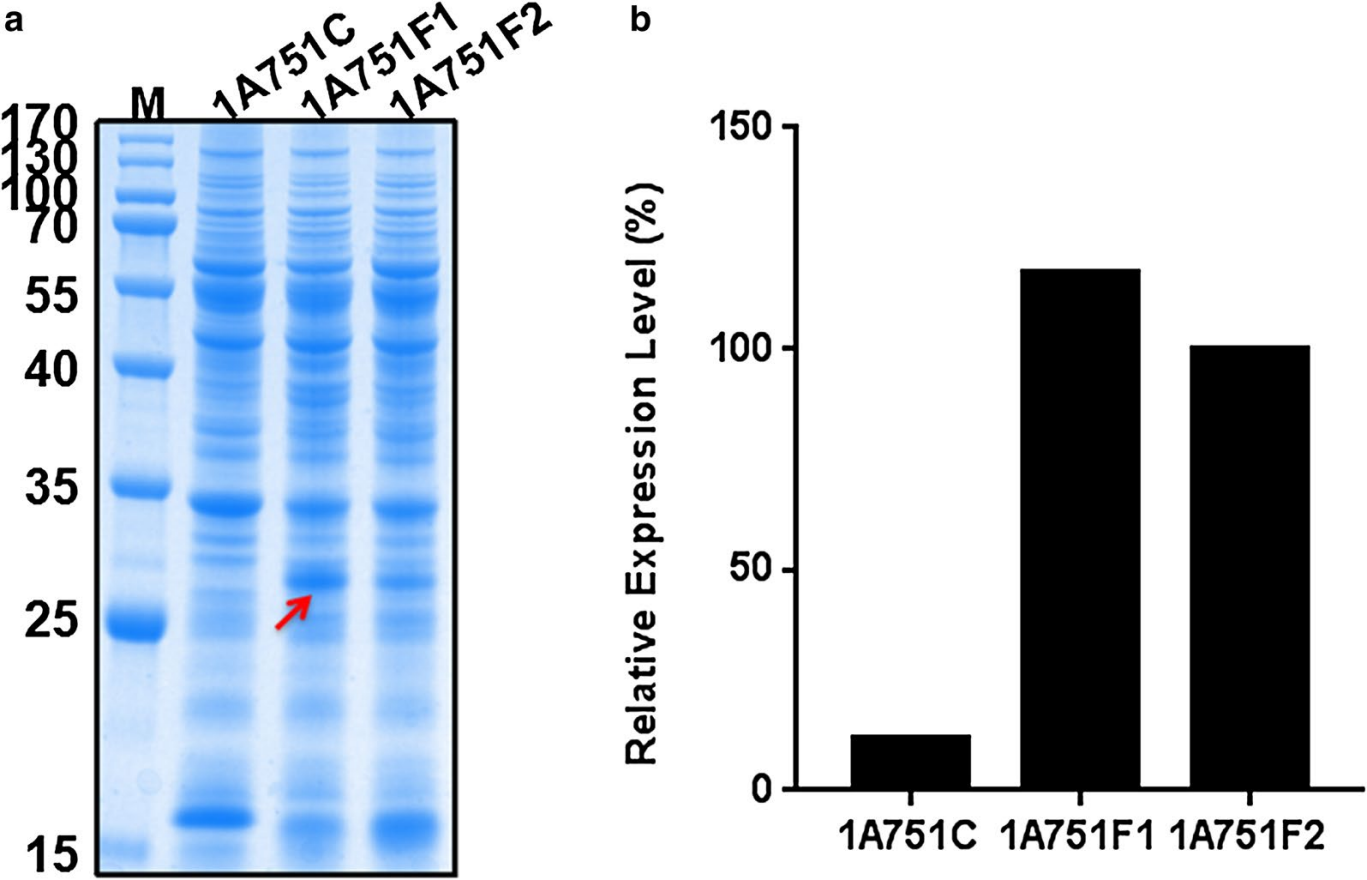
Comparison of rhFGF21 gene expression with different promoters. **a** SDS-PAGE analysis of rhFGF21 expressed in the cytoplasm of *B. subtilis* following 24 h incubation. The molecular weight of mature rhFGF21 is about 27 kDa. Column M: molecular weight marker. The strain *B. subtilis* 1A751C served as negative control. **b** The relative densitometric analysis for rhFGF21 expression level in different *B. subtilis* strains

The plasmid pMA5, an *E. coli/B. subtilis* shuttle vector which is derived from pUB110 was used for expression of the rhFGF21 protein. To achieve a suitable transcription level of the target protein, the originate constitutive promoter P_*hpaII*_ [26] in plasmid pMA5 was replaced by a maltose-inducible promoter P_*malA*_ [27], generating pMATE plasmid. Meanwhile, pMA5 with promoter P_*hpaII*_ was also used for constructing the expression plasmid of rhFGF21. The nucleic acid sequence of rhFGF21 with C-terminal His-tag was codon optimized and synthesized (GENEWIZ Suzhou, China) for efficient expression in *B. subtilis*. To achieve secretory expression of rhFGF21 protein in *B. subtilis*, a widely used signal peptide SP_*pelB*_ sequence from *B. subtilis* was cloned upstreaming of the rhFGF21 gene in plasmid pMA5 and pMATE, generating plasmid pMATEF and pMA5F. Then the contructs pMATEF, pMA5F and the empty plasmid pMATE were transformed into *B. subtilis* 1A751, resulting in three recombinant strains 1A751F1, 1A751F2 and 1A751C. After 24 h flask fermentation supplied with 0.2% (w/v) maltose, cultures of above strains were harvested to perform SDS-PAGE analysis. The SDS-PAGE results (Fig. 2) indicated that both two constructs (pMATEF and pMA5F) can successfully express rhFGF21 within cells, which reveal a distinct band (27 kDa) in good agreement with the theoretical molecular weight of the precursor of rhFGF21 before the cleavage of signal peptide. Moreover, the target band from strain 1A751F1 was significantly broader than that from strain 1A751F2, which is consistent with the strength of the promoters reported in a previous study [28]. Thus, we speculated that the expression of rhFGF21 may be consistent with the strength of promoters, and that the strong promoter P_*malA*_ may have an edge on P_*hpaII*_. However, the expression level of rhFGF21 still has giant improving space for further optimization. Thus, we attempted other strategies to improve the production of protein rhFGF21 in *B. subtilis*.

### Enhancing translation efficiency of rhFGF21 in *B. subtilis* by introducing a mini-cistron cassette

**Fig. 3:**
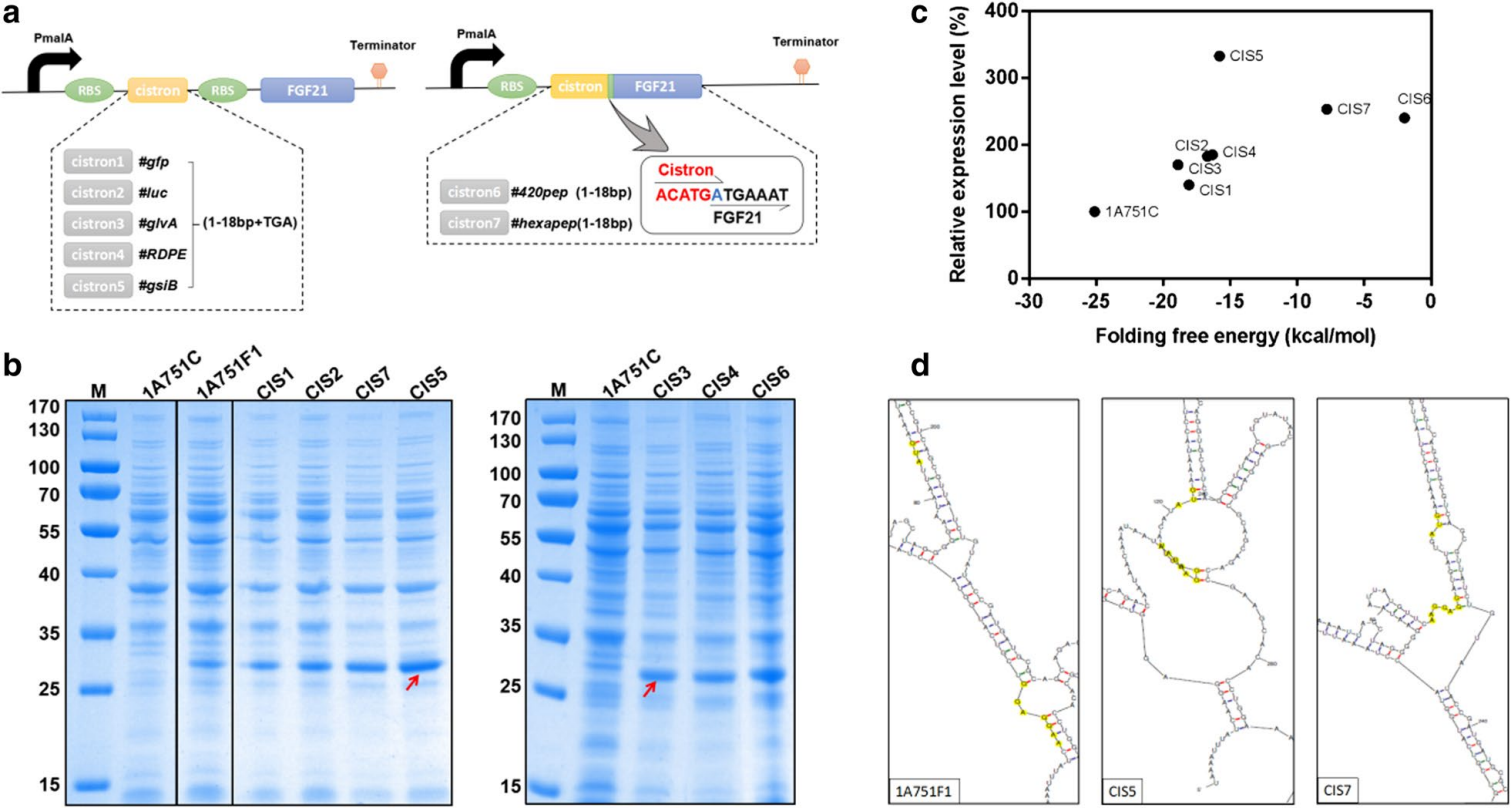
Comparison of improvement efficiency of different mini-cistron cassettes. **a** Schematic representation of the different mini-cistrons cassettes. P_*malA*_ is the rhFGF21′s promoter in *B. subtilis*, RBS, ribosome binding site. **b** SDS-PAGE analysis of seven recombinant strains (CIS1 (pMATEFc1), CIS2 (pMATEFc2), CIS3 (pMATEFc3), CIS4 (pMATEFc4), CIS5 (pMATEFc5), CIS6 (pMATEFc6) and CIS7 (pMATEFc7)) and their TCP expression in *B. subtilis* following 24 h incubation. The molecular weight of mature rhFGF21 is about 27 kDa. Column M: molecular weight marker. The strains 1A751C and 1A751F1 served as negative and positive controls. **c** The scatter plot of the relationship between relative expression level and the folding free energy of rhFGF21. The relative expression level of rhFGF21 was predicted using Bio-Rad Image Lab Software 5.2.1. The relative expression of the seven recombinant strains was compared with that of the strain 1A751F1 (pMATEF). The folding free energy of rhFGF21 was predicted using the mfold Web Server. **d** Partial views of mRNA secondary structures of the full-length transcripts corresponding to the rhFGF21 protein, predicted using the mfold Web Server. The highlighted areas are the initiation codons and RBS sequences of different strains

Low-level production of target proteins is frequently caused by rare codon usage [29, 30], weak promoters [31], or poor translation initiation [32]. In order to reduce rare codon usage, the coding sequence of rhFGF21 was optimized for *B. subtilis* in our expression trial. We also selected the strong promoter P_*malA*_ for the expression of rhFGF21. Previous studies showed the 5′ Untranslated Region (5′UTR) of a poorly expressed heterologous protein may form a stable and complex mRNA secondary structure, which can bury the ribosome binding site (RBS) region and the ATG start codon of the target protein, resulting in a decrease in translation initiation efficiency [33]. Structure of mini-cistron cassettes could reduce locally stabilized mRNA secondary structures of the downstream target gene and result in an improvement of translation initiation efficiency [34–37]. Thus, to destabilize any potential stem-loop secondary structures in the rhFGF21 mRNA sequence, we designed seven auxiliary mini-cistron cassettes and introduced them by inserting mini open reading frames (ORF) upstream of the rhFGF21 gene. As shown in Fig. 3a, the first five mini-cistron cassettes contain a short ORF deriving from the first 6 amino acids of several highly expressed proteins (GFP, Luciferase, GlvA, RDPE [17] and GsiB) in *B. subtilis*, followed by a stop codon TGA and a RBS, respectively. The two remaining mini-cistron cassettes originate from the polypeptides (the first 6 amino acids) 420pep and hexapep [38], which were translationally coupled to the coding sequence of rhFGF21 by an overlap at the adenine nucleotide between the stop and start sites- TGATG [36, 39]. A RBS sequence (AAGGAGG) was embedded within the sequences of 420pep and hexapep respectively. By transforming these expression vectors with mini-cistron cassettes into *B. subtilis* 1A751, seven recombinant strains were generated as follows: CIS1 (pMATEFc1), CIS2 (pMATEFc2), CIS3 (pMATEFc3), CIS4 (pMATEFc4), CIS5 (pMATEFc5), CIS6 (pMATEFc6) and CIS7 (pMATEFc7). To evaluate the translation optimization of the seven mini-cistron cassettes, the new recombinant strains were compared to the control strains 1A751C and 1A751F1 in 250 mL Erlenmeyer flask fermentation under 0.2% (w/v) maltose induction. Then we compared the expression level of rhFGF21 in different strains by SDS-PAGE analysis. As shown in Fig. 3b, compared to *B. subtilis* 1A751F1, the seven recombinant strains all showed increased expression level of rhFGF21, which indicates that the mini-cistron cassettes definitely have a positive effect on rhFGF21 expression. To precisely compare the differences between the seven recombinant strains, we estimated the relative expression level of rhFGF21 by densitometry analysis of the electrophoresis bands via the Bio-Rad Image Lab Software 5.2.1, as shown in Additional file 1: Figure S1. The relative expression level of the recombinant strain CIS5 was significantly higher than that of other strains, which was increased to approximately 3 times over the originate strain 1A751F1. These results indicated that cistron5 (GsiB) is an efficient mini-cistron cassette for enhancing the production of rhFGF21 in *B. subtilis*.

Previous study also proved that the expression of nrIL-24 in *E. coil* was increased to 26% of the total cellular protein from being barely initially detectable via optimizing the secondary structure of the translation initiation region and increasing the Gibbs free energy of the region [40]. Therefore we presumed that, by introducing of different mini-cistron cassettes upstream of the target gene, the changes of Gibbs free energy in the 5′ UTR mRNA secondary structure may contribute to the increase of translation initiation efficiency, result in the improving of expression level of rhFGF21. So we measured the corresponding minimum free energy (ΔG) of mRNA secondary structures formed by the codons starting at 135 bp from the transcriptional start site of the native rhFGF21 and the engineered mini-cistron cassettes with rhFGF21 using the mfold Web Server [41]. As shown in Fig. 3c, we found a positive correlation between the relative expression level of rhFGF21 and the corresponding ΔG in different *B. subtilis* strains. To some extent, as the ΔG increased, the relative expression level of rhFGF21 increased as well. This indicates that increasing the ΔG of the mRNA secondary structure may enhance the expression level of rhFGF21. In addition, we monitored the corresponding mRNA secondary structures of the cistron5 cassette and cistron7 cassette, which were efficient in enhancing the expression level of rhFGF21 (Fig. 3d). The predicted mRNA secondary structures demonstrated the transcripts of the two mini-cistron cassettes provided easy access to the start codons by the translation machinery. The RBS sequences of the two mini-cistron cassettes were more easily exposed and this potentially increased ribosome recruitment, when compared with the native rhFGF21 sequence. Thus, we speculated that the translation efficiency of rhFGF21 is linked to the stability of the mRNA secondary structure.

### Enhancing soluble expression of rhFGF21 by overexpression of chaperones

**Fig. 4:**
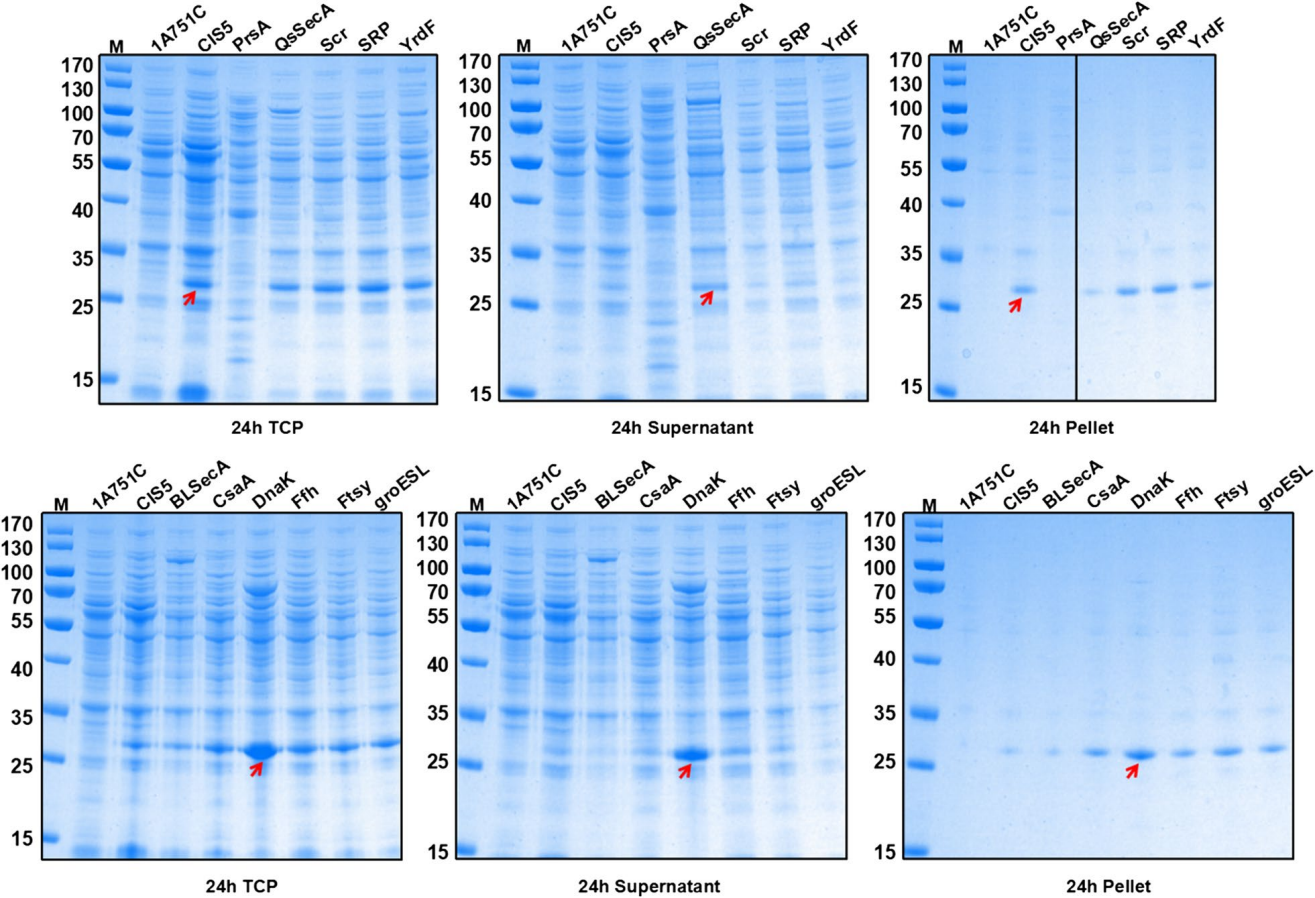
SDS-PAGE analysis of rhFGF21 expression following ultrasonication. Total cell protein (TCP), supernatant, and pellet fractions from *B. subtilis* strains that overexpressed different chaperones at incubation of 24 h were run. Strains *B. subtilis* CHAP1, CHAP2, CHAP3, CHAP4, CHAP5, CHAP6, CHAP7, CHAP8, CHAP9, CHAP10, and CHAP11 overexpressed *blsecA*, *csaA*, *dnak*, *ffh*, *ftsY*, *groESL* operon, *prsA*, *qssecA*, *scr*, *SRP,* and *yrdF*, respectively. The molecular weight of mature rhFGF21 is about 27 kDa. Column M: molecular weight marker. The strains 1A751C and CIS5 served as negative and positive controls

Molecular chaperones can assist the folding of newly synthesized proteins to the native state and provide a quality control system assists in refolding misfolded and aggregated proteins [42]. Overexpression of one or more chaperones has been shown in a number of studies to assist the folding and expression of recombinant proteins [43, 44]. To further increase the solubility of rhFGF21 in *B. subtilis*, eleven recombinant strains, CHAP1 (*blsecA*), CHAP2 (*csaA*), CHAP3 (*dnak* operon), CHAP4 (*ffh*), CHAP5 (*ftsY*), CHAP6 (*groESL* operon), CHAP7 (*prsA*), CHAP8 (*qssecA*), CHAP9 (*scr*), CHAP10 (*SRP*), and CHAP11 (*yrdF*) (Table 1) were constructed by transforming different integration vectors (Table 2) containing the corresponding chaperones into *B. subtilis* CIS5. These eleven chaperone genes were overexpressed under the control of a strong constitutive promoter P_*grac*_ and were integrated into the *B. subtilis* chromosome at *amyE* locus via double crossing-over [16, 18]. These chaperones could mediate target protein folding, decrease aggregation, and maintain pre-proteins in translocation-competent conformations [45]. The expression level of rhFGF21 was evaluated by flask fermentation adding 0.2% (w/v) maltose as inducer, and the 1A751C and CIS5 strains served as controls. SDS-PAGE analysis was performed to compare the production levels of rhFGF21 (Fig. 4). Compared with *B. subtilis* CIS5, the recombinant strain CHAP3 overexpressing the *dnak* operon drastically enhanced the soluble expression up to 987%, which achieved the highest expression of rhFGF21 both in total cell proteins and soluble proteins. In addition, overexpression of the chaperone CHAP2 (*csaA*), CHAP4 (*ffh*), CHAP5 (*ftsY*), CHAP8 (*qssecA*), and CHAP10 (*SRP*) also showed a positive effect on protein solubility, but the improvements were not as significant as that observed in CHAP3 (*dnak* operon). Therefore, we concluded that DnaK is the most efficient chaperone for rhFGF21 soluble expression in *B. subtilis*.

**Table 1:** Strains used in this study

Strains	Genotype and/or relevant characteristic (s)	Source
*E. coli* DH5α	F−Δ*lac*U169(Ø80d *lac*ZΔM15) *sup*E44 *hsd*R17 *rec*A1 *gyr*A96 *end*A1 *thi*-1 *rel*A1	Invitrogen
*B. subtilis *1A751	*egl*S∆102 *bgl*T/*bgl*S∆EV *apr*E *npr*E *his*	BGSC
Kno6	*egl*S∆102 *bgl*T/*bgl*S∆EV *apr*E *npr*E *his* ∆*bpr*∆*epr*∆*HtrA*∆*mpr*∆ *nprB*∆*vpr*	This work
1A751C	1A751 containing pMATE; Km^r^	This work
1A751F1	1A751 containing pMATEF; Km^r^	This work
1A751F2	1A751 containing pMA5F; Km^r^	This work
CIS1	1A751 containing pMATEFc1; Km^r^	This work
CIS2	1A751 containing pMATEFc2; Km^r^	This work
CIS3	1A751 containing pMATEFc3; Km^r^	This work
CIS4	1A751 containing pMATEFc4; Km^r^	This work
CIS5	1A751 containing pMATEFc5; Km^r^	This work
CIS6	1A751 containing pMATEFc6; Km^r^	This work
CIS7	1A751 containing pMATEFc7; Km^r^	This work
CHAP1	CIS5 containing pDF1; Cm^r^, Km^r^	This work
CHAP 2	CIS5 containing pDF2; Cm^r^, Km^r^	This work
CHAP 3	CIS5 containing pDF3; Cm^r^, Km^r^	This work
CHAP 4	CIS5 containing pDF4; Cm^r^, Km^r^	This work
CHAP 5	CIS5 containing pDF5; Cm^r^, Km^r^	This work
CHAP 6	CIS5 containing pDF6; Cm^r^, Km^r^	This work
CHAP 7	CIS5 containing pDF7; Cm^r^, Km^r^	This work
CHAP 8	CIS5 containing pDF8; Cm^r^, Km^r^	This work
CHAP 9	CIS5 containing pDF9; Cm^r^, Km^r^	This work
CHAP 10	CIS5 containing pDF10; Cm^r^, Km^r^	This work
CHAP 11	CIS5 containing pDF11; Cm^r^, Km^r^	This work
CHAP3R1	CHAP3 containing pMATEFc5R1; Cm^r^, Km^r^	This work
CHAP3R2	CHAP3 containing pMATEFc5R2; Cm^r^, Km^r^	This work
CHAP3R3	CHAP3 containing pMATEFc5R3; Cm^r^, Km^r^	This work
Kno6cs	Kno6 containing pMATEFc5; Km^r^	This work
Kno6cf	Kno6 containing pDF3, pMATEFc5; Cm^r^, Km^r^	This work
Kno6k	Kno6 containing pDF3; Cm^r^	This work
Kno6csp 1	Kno6k containing pMATEF1; Cm^r^, Km^r^	This work
Kno6csp 2	Kno6k containing pMATEF2; Cm^r^, Km^r^	This work
Kno6csp 3	Kno6k containing pMATEF3; Cm^r^, Km^r^	This work
Kno6csp 4	Kno6k containing pMATEF4; Cm^r^, Km^r^	This work
Kno6csp 5	Kno6k containing pMATEF5; Cm^r^, Km^r^	This work
Kno6csp 6	Kno6k containing pMATEF6; Cm^r^, Km^r^	This work
Kno6csp 7	Kno6k containing pMATEF7; Cm^r^, Km^r^	This work
Kno6csp 8	Kno6k containing pMATEF8; Cm^r^, Km^r^	This work
Kno6csp 9	Kno6k containing pMATEF9; Cm^r^, Km^r^	This work
Kno6csp 10	Kno6k containing pMATEF10; Cm^r^, Km^r^	This work
Kno6csp 11	Kno6k containing pMATEF11; Cm^r^, Km^r^	This work

**Table 2:** Plasmids used in this study

Plasmids	Genotype and/or relevant characteristic(s)	Source
pMA5	*E. coli*/*B. subtilis* shuttle vector, P_*HpaII*_, Ap^r^, Km^r^	BGSC
pMATE	pMA5 derivative, P_*malA*_	This work
pMA5F	pMA5 derivative, SP_*pelB*_-*rhfgf21*	This work
pMATEF	pMATE derivative, SP_*pelB*_-*rhfgf21*	This work
pMATEFc1	pMATEF derivative, cistron1	This work
pMATEFc2	pMATEF derivative, cistron2	This work
pMATEFc3	pMATEF derivative, cistron3	This work
pMATEFc4	pMATEF derivative, cistron4	This work
pMATEFc5	pMATEF derivative, cistron5	This work
pMATEFc6	pMATEF derivative, cistron6	This work
pMATEFc7	pMATEF derivative, cistron7	This work
pDF	Integration vector, pDL derivative, P_grac101_, Ap^r^, Cm^r^	Lab stock
pDF-d	pDF derivative, *dsbA*	This work
pDF1	pDF derivative, *blsecA*	This work
pDF2	pDF derivative, *csaA*	This work
pDF3	pDF derivative, *dnak*	This work
pDF4	pDF derivative, *ffh*	This work
pDF5	pDF derivative, *ftsY*	This work
pDF6	pDF derivative, *groESL*	This work
pDF7	pDF derivative, *prsA*	This work
pDF8	pDF derivative, *qssecA*	This work
pDF9	pDF derivative, *scr*	This work
pDF10	pDF derivative, *SRP*	This work
pDF11	pDF derivative, *yrdF*	This work
pMATEFc5R1	pMATEFc5 derivative, *rhfgf21*(*L98R*)	This work
pMATEFc5R2	pMATEFc5 derivative, *rhfgf21*(*P171A*)	This work
pMATEFc5R3	pMATEFc5 derivative, *rhfgf21*(*P171G*)	This work
pMATEF1	pMATEFc5 derivative, SP_*phoD*_-*rhfgf21*	This work
pMATEF2	pMATEFc5 derivative, SP_*pel*_-*rhfgf21*	This work
pMATEF3	pMATEFc5 derivative, SP_*ywbN*_-*rhfgf21*	This work
pMATEF4	pMATEFc5 derivative, SP_*lipA*_-*rhfgf21*	This work
pMATEF5	pMATEFc5 derivative, SP_*protA*_-*rhfgf21*	This work
pMATEF6	pMATEFc5 derivative, SP_*ywmC*_-*rhfgf21*	This work
pMATEF7	pMATEFc5 derivative, SP_*dacB*_-*rhfgf21*	This work
pMATEF8	pMATEFc5 derivative, SP_*nprE*_-*rhfgf21*	This work
pMATEF9	pMATEFc5 derivative, SP_*yddT*_-*rhfgf21*	This work
pMATEF10	pMATEFc5 derivative, SP-_*yoqm*_*rhfgf21*	This work
pMATEF11	pMATEFc5 derivative, SP-_*yvce*_*rhfgf21*	This work

### Enhancing stability of secreted rhFGF21 by knocking out extracellular proteases

**Fig. 5:**
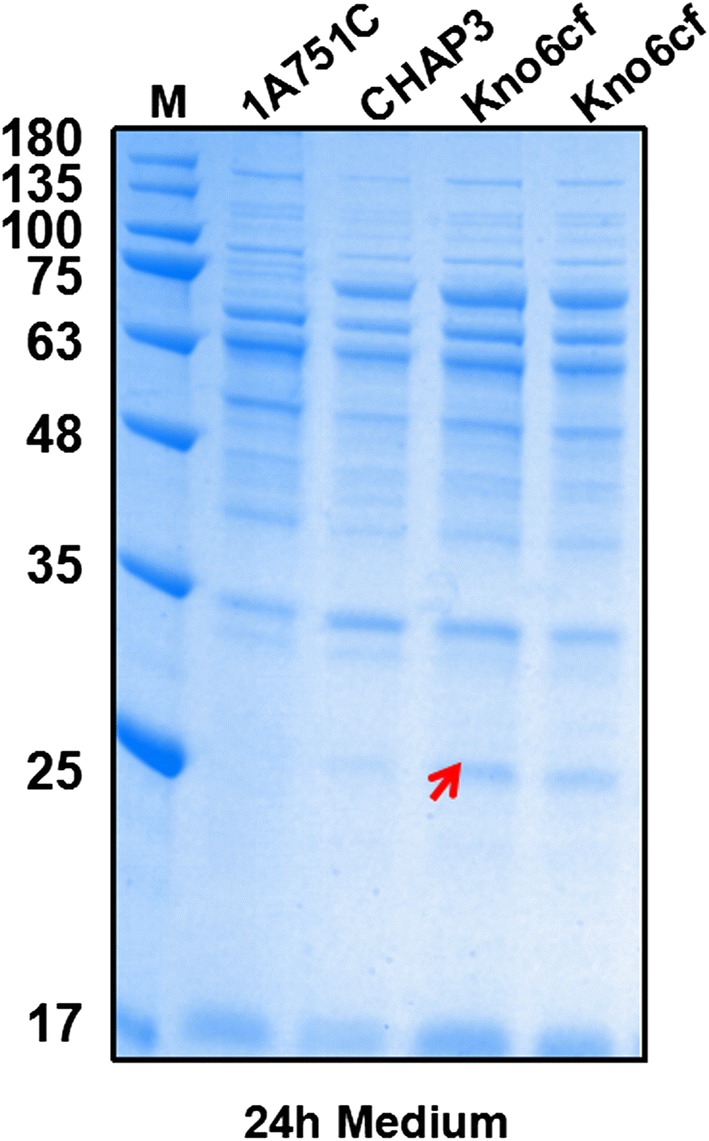
SDS-PAGE analysis of expression of six extracellular proteases deficient strain Kno6cf in culture medium following 24 h incubation. The molecular weight of mature rhFGF21 is about 25 kDa. Column M: molecular weight marker. The strain 1A751C served as negative control

During the post-log phase in *B. subtilis* growth, the bacteria express and secrete large amounts of protease, making secreted heterologous proteins difficult to accumulate due to protease hydrolysis [46, 47]. Therefore, knocking out extracellular proteases was deemed to be crucial for heterologous protein expression and secretion in *B. subtilis*. Since the host strain *B. subtilis* 1A751 is already deficient in two major extracellular proteases (*nprE* and *aprE*) [17], we knocked out the remaining six genes that express extracellular proteases in *B. subtilis* 1A751: *bpr*, *epr*, *htrA*, *mpr*, *nprB*, and *vpr,* to protect the target protein rhFGF21 from protease degradation. Then, we chose the pDF3 and pMATEFc5 plasmids and transformed them into this protease-deficient strain Kno6 using the “Paris Method” which is described in “Methods” section, resulting in the recombinant strain Kno6cf. After 24 h flask fermentation supplied with 0.2% (w/v) maltose, cultures of Kno6cf strain were harvested to perform SDS-PAGE to determine rhFGF21 secretion level. The 1A751C and CHAP3 strains served as negative and positive controls, respectively. As shown in Fig. 5, compared to the CHAP3 strain, the extracellular proteases deficient strain Kno6cf successfully secreted the mature rhFGF21 protein (25 KDa) processing from the rhFGF21 precursor, while no target protein was observed in the CHAP3 strain culture medium. These results illustrate that the target protein rhFGF21 is prone to be degraded by extracellular proteases and that a protease-deficient strain is necessary for maintaining the stability of mature secreted rhFGF21.

### Enhancing secretion efficiency of rhFGF21 by screening different signal peptides

**Fig. 6:**
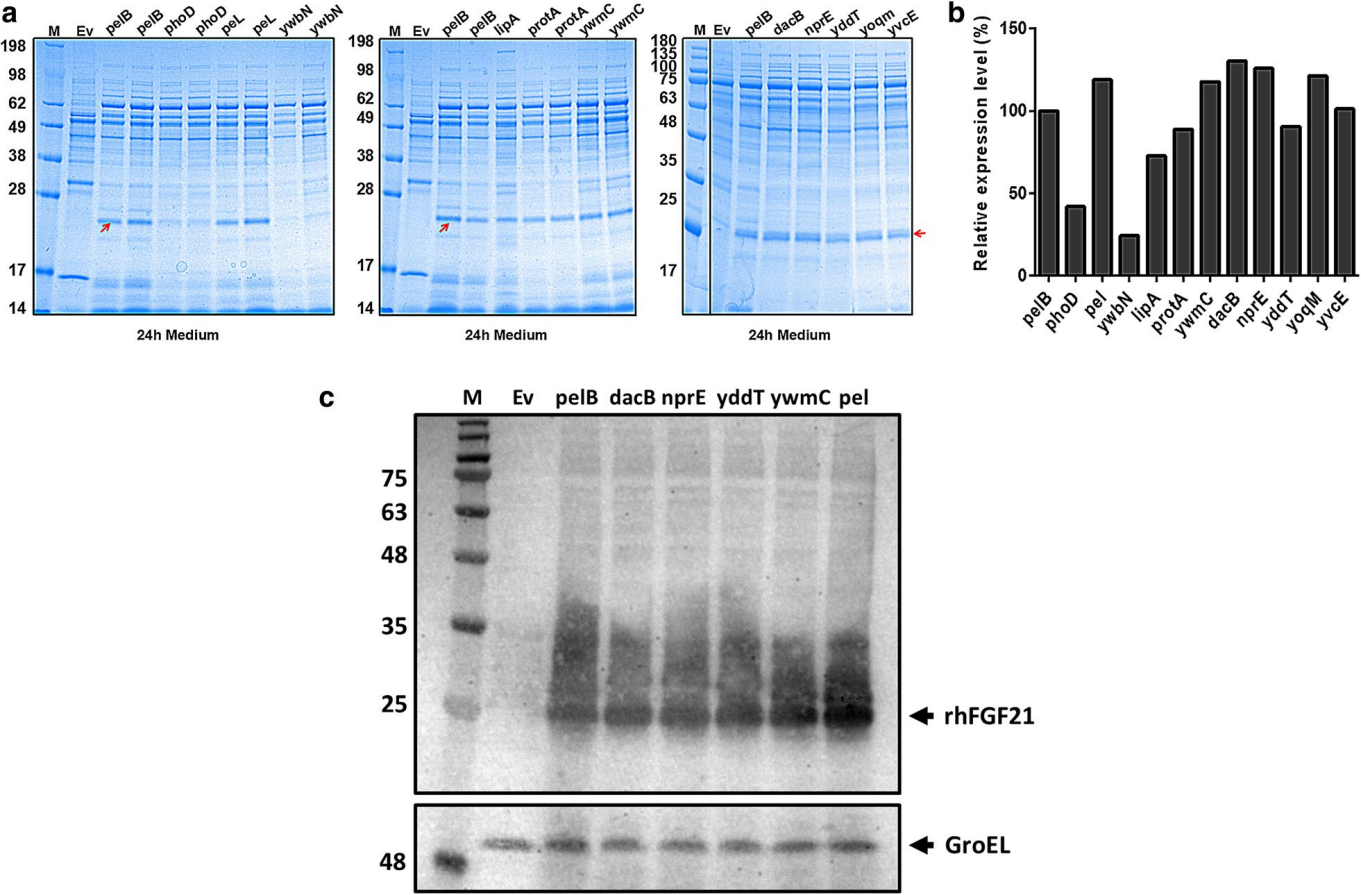
Comparison of efficiency of different signal peptides. **a** SDS-PAGE analysis of different signal peptides in culture medium following incubation for 24 h. The molecular weight of mature rhFGF21 is about 27 kDa. Column M: molecular weight marker. Column Ev: plasmids of pMATE of strain 1A751C served as negative control. **b** The relative densitometric analysis for rhFGF21 secreted by different *B. subtilis* strains. **c** Western blotting analysis of the secreted rhFGF21 protein with different signal peptides in *B. subtilis*

In general, to secrete rhFGF21 directly into the extracellular medium, it is necessary to screen for suitable signal peptides that function well in *B. subtilis*. In *B. subtilis*, the export of protein is generally accomplished by the Sec or Tat secretion system. When a target protein is fused to an N-terminal secretion signal known as a signal peptide, it can be recognized and translocated by the Sec or Tat machinery through the membrane into the extracellular medium [48]. To analyze the effect of different signal peptides, eleven signal peptides were screened in *B. subtilis* (Table 3), which were reported to highly direct the secretion of different enzymes [16]. They belong to the general secretory (Sec) pathway (SP_*pel*_, SP_*lipA*_, SP_*protA*_, SP_*ywmC*_, SP_*dacB*_, SP_*nprE*_, SP_*yddT*_, SP_*yoqM*_, SP_*yvcE*_) and the twin-arginine translocation (Tat) pathway (SP_*phoD*_, SP_*ywbN*_) [49, 50]. Different signal peptide fragments were amplified and cloned between P_*malA*_ and the rhFGF21 gene in pMATEFc5. These plasmids were then transformed into the recombinant strain Kno6k, which overexpressed the *dnaK* operon, resulting in the following strains: Kno6csp1 (SP_*phoD*_), Kno6csp2 (SP_*pel*_), Kno6csp3 (SP_*ywbN*_), Kno6csp4 (SP_*lipA*_), Kno6csp5 (SP_*protA*_), Kno6csp6 (SP_*ywmC*_), Kno6csp7 (SP_*dacB*_), Kno6csp8 (SP_*nprE*_), Kno6csp9 (SP_*yddT*_), Kno6csp10 (SP_*yoqM*_) and Kno6csp11 (SP_*yvcE*_). For secretory expression test, different strains were grown in 250-mL Erlenmeyer flasks containing 25 mL SR medium at 37 °C for 24 h, maltose was added to a final concentration of 0.2% (w/v) to induce the expression of rhFGF21. The recombinant strain Kno6cf (pMATEFc5 and pDF3) that the rhFGF21 was leading by a signal peptide SP_*pelB*_ was used as a positive control. We harvested the fermentation medium to obtain samples for SDS-PAGE analysis, and the results are shown in Fig. 6a. We found most of the recombinant strains could secrete rhFGF21 into the culture medium, and a band in accordance with the theoretical molecular weight of the mature protein was observed. The relative expression analysis of rhFGF21 was estimated using the Bio-Rad Image Lab Software 5.2.1, as shown in Fig. 6b. Compared to the other eleven signal peptides, a Sec pathway signal peptide SP_*dacB*_ (Kno6csp7) was the most efficient one for protein secretion. But the two Tat pathway signal peptides (SP_*phoD*_, SP_*ywbN*_) represented the lowest secretion efficiencies, which proved that Tat signal peptides could not successfully lead the rhFGF21 protein secreted to the extracellular medium, and the Tat-pathway is not suitable for our target protein rhFGF21 in *B. subtilis*. Based on the above observations, we concluded that rhFGF21 was suitable for secretion using the signal peptide SP_*dacB*_ from the Sec-pathway. As shown in Fig. 6c, the secreted rhFGF21 proteins with His-tag from different strains were further analyzed by western blotting using anti-His antibody as a probe. We examined the secretion fractions of different signal peptides (SP_*pelB*_, SP_*dacB*_, SP_*nprE*_, SP_*yddT*_, SP_*ywmC*_ and SP_*pel*_) and proved that all of them were well-processing rhFGF21after secreted from *B. subtilis*.

**Table 3:** Comparison of all screened signal sequences used in this study

No.	SP	Sequences	Length (aa)	Type	Origins	D value
1	pelB	MKYLLPTAAAGLLLLAAQPAMA	22	Sec	*E. coli*	0.512
2	phoD	MAYDSRFDEWVQKLKEESFQNNTFDRRKFIQGAGKIAGLSLGLTIAQSVGAFEVNA	56	Tat	*B. subtilis*	0.245
3	pel	MKKVMLATALFLGLTPAGANA	21	Sec	*B. subtilis*	0.258
4	ywbN	MSDEQKKPEQIHRRDILKWGAMAGAAVA	28	Tat	*B. subtilis*	0.526
5	lipA	MKFVKRRIIALVTILMLSVTSLFALQPSAKAA	32	Sec	*B. subtilis*	0.332
6	protA	MKKKNIYSIRKLGVGIASVTLGTLLISGGVTPAANA	36	Sec	*S. aureus*	0.500
7	ywmC	MKKRFSLIMMTGLLFGLTSPAFA	23	Sec	*B. subtilis*	0.358
8	dacB	MRIFKKAVFVIMISFLIATVNVNTAHA	27	Sec	*B. subtilis*	0.478
9	nprE	MGLGKKLSVAVAASFMSLSISLPGVQA	27	Sec	*B. subtilis*	0.225
10	yddT	MRKKRVITCVMAASLTLGSLLPAGYASA	28	Sec	*B. subtilis*	0.282
11	yoqM	MKLRKVLTGSVLSLGLLVSASPAFA	25	Sec	*B. subtilis*	0.339
12	yvcE	MRKSLITLGLASVIGTSSFLIPFTSKTASA	30	Sec	*B. subtilis*	0.322

### Characterization of the synthesized and secreted rhFGF21 protein

To analyze whether the secreted rhFGF21 was properly processed by signal peptidase in *B. subtilis*, rhFGF21 protein with C-terminal his-tag was purified with Ni–NTA resin-based affinity chromatography and analyzed with LC–MS. SDS-PAGE analysis (Additional file 2: Figure S2) revealed the purified secreted rhFGF21 from *B. subtilis* showed a similar molecular weight (about 25 kDa) with the rhFGF21 standard sample provided by Nova nordisk. LC–MS results (Additional file 3: Figure S3) indicated that the purified protein showed a spectral peak at m/z 20231, which was consistent with the value of intact mature hFGF21 with his-tag (20233), corresponding to the ion of rhFGF21 detected with just a 0.009% error. This is a reasonable error at high molecular weights in MS analysis, meaning that signal peptide has been properly removed in the secreted mature rhFGF21 protein. The mismatch of molecular weight between SDS-PAGE and MS analysis may caused by the imprecise rough MW determination by SDS-PAGE that the mobility of band could be affected by different states (reduced or non-reduced) of protein with disulfide bonds [51]. So MS analysis is a more reliable method for determination of MW.

To evaluate the bioactivity of the secreted rhFGF21 from *B. subtilis* Kno6cf, we examined phosphorylation of ERK, an event known to be downstream of FGF receptor activation [52]. The rhFGF21 protein purified from Kno6cf strain was evaluated head-to-dead with rhFGF21 standard sample in an ERK1/2 phosphorylation assay in HEK-hBKL cells with stable over-expression of βKlotho (Klb), a critical co-factor for FGF21 action [53]. Both, rhFGF21 purified from *B. subtilis* and rhFGF21 standard sample stimulated ERK1/2 phosphorylation in HEK-hBKL cells (Additional file 4: Figure S4). In this assay, purified rhFGF21 from *B. subtilis* and rhFGF21 sample demonstrated comparable potency (Additional file 5: Table S1).

## Discussion

To our knowledge, this is the first report of highly efficient expression and secretion of rhFGF21 in *B. subtilis.* As shown in Fig. 1, to express and secrete the heterologous protein rhFGF21 in *B. subtilis*, we attempted different strategies to address the potential bottlenecks in protein expression and secretion. Finally, these strategies were combined to improve soluble expression and secretion level of rhFGF21.

Using a high copy-number plasmid combined with a strong promoter was a good initial strategy for protein production. We selected two promoters: a constitutive promoter P_*hpaII*_ and a maltose-inducible promoter P_*malA*_, which are widely used strong promoters in *B. subtilis* protein expression [16, 27, 54]. Maltose is a low-cost inducer and also serves as a fermentation carbon source. In order to offset disturbances in carbon sources, we kept the same concentration of maltose in the fermentation media of different recombinant strains. Based on our findings, P_*malA*_ proved to be the most efficient for rhFGF21 production. We showed that the use of a strong promoter results in high transcription levels, which enhances the expression level of rhFGF21.

Ishida et al. reported that poor expression of genes can be due to inefficient translation initiation, and they introduced an overlapping leader ORF to activate the translation of a downstream gene through efficient translation re-initiation to improve the expression of the target protein [37]. In order to further increase the production of rhFGF21, different types of auxiliary mini-cistron cassettes were designed to disrupt the locally stabilized mRNA secondary structure around the RBS and initiation codon, and to enable efficient enhancement of translation initiation. The RBS and the start codon, if sequestered in a structured region of the mRNA, can decrease accessibility for ribosomes and diminish translation [23]. Our findings indicate that the introduced mini-cistron cassette led to a more open conformation and the ΔG of mRNA secondary structures was increased. We believe that reducing the stability of mRNA secondary structures around the RBS and initiation codon destabilized the stem-loop structure and reduced the corresponding ΔG, which led to more exposed RBS sequences and more easily accessible initiation codons. Ribosome recruitment had the potential to be increased, which may have resulted in the translational enhancement of the target protein rhFGF21. We acknowledge that this hypothesis needs further experimental testing as there are multifactorial interactions at play when correlating protein expression with mRNA folding, such as ribosome binding, translation speed, translation abortion, and helicase activity disrupting secondary structures [34, 35]. However, the idea is worth exploring, as the mini-cistron cassette approach may be useful for the expression of other heterologous proteins in *B. subtilis*, including cytokines and enzymes.

The production of rhFGF21 was mostly in inclusion bodies in *B. subtilis*. In an effort to improve the soluble expression of the rhFGF21 protein in *B. subtilis*, we evaluated the overexpression of individual chaperones using 11 genes or gene operons involved in protein folding or translocation, and found that the overexpression of the *dnak* operon markedly improved the production of rhFGF21 (Fig. 4). According to our experimental results, the chaperone encoded by the *dnak* operon can assist in the folding of newly synthesized proteins to their native state in *B. subtilis*. Our results indicate the bottleneck of low soluble protein expression, as it pertains to rhFGF21, was resolved by overexpression of the chaperone encoded by the *dnak* operon. In addition, individual overexpression of *blsecA*, *csaA*, *ffh*, *ftsY*, *groESL* operon, *prsA*, *qssecA*, *scr*, *SRP*, and *yrdF* moderately or marginally improved the production of soluble rhFGF21. From these results, we suspect that the expression of soluble rhFGF21 would further be increased by some combinatorial overexpression of several chaperones.

In *B. subtilis*, there are several proteases secreted into the extracellular medium and the expression of soluble excreted heterologous proteins is degraded due to protease hydrolysis. Therefore, the accumulation of extracellular rhFGF21 was achieved by knocking out the genes encoding extracellular proteases in *B. subtilis* 1A751. In addition, eleven signal peptides from sec- or tat-pathways were screened to enhance the secretion of rhFGF21 in *B. subtilis.* The results indicate that rhFGF21 tends to be secreted through the Sec-pathway signal peptide SP_*dacB*_. However, a large quantity of precursor protein remained within the cytoplasm. We optimized transport proteins by individual overexpression of 10 genes and found that the secretion of rhFGF21was not increased significantly (data not shown). This result implied that individual overexpression of transport proteins is not sufficient to further increase the secretion of rhFGF21, which remains a challenging task that testing the effect of combinatorial overexpression of several transport proteins in the future.

Following the implementation of the above strategies, expression of soluble rhFGF21 achieved a relatively high level in the cytoplasm, but there was an obvious secretion bottleneck hindering transport of the rhFGF21 protein into the extracellular milieu. Although we attempted to optimize several signal peptides and overexpress transport proteins, the secretion level of rhFGF21 was still rather low. We also designed some additives to the culture media, such as alcohol, glycine, betaine, and sorbitol, which were supposed to prevent the strain from osmotic pressure or assist the cell permeability, but protein secretion was not obviously enhanced (data not shown). We suspect that it could relate to disulfide bond formation of and the transport mechanism in *B. subtilis* cells, and we will further study the problem in the future.

## Conclusions

We have constructed a maltose-inducible expression system for the expression and secretion of the heterologous protein rhFGF21 in *B. subtilis.* Two strong promoters, P_*hpaII*_ and P_*malA*_ were tested for improving the expression level of our target protein, and promoter P_*malA*_ exhibits an improvement of the expression level of the rhFGF21 gene. A mini-cistron cassette (from GsiB) was intercalated upstream of rhFGF21 in the expression vector to reduce putative locally stabilized mRNA secondary structure and enhance translation initiation, thus increasing the expression of the rhFGF21 protein. Overexpression of the DnaK chaperone further increased the soluble expression of rhFGF21 significantly. The extracellular protease-deficient expression strain *B. subtilis* Kno6cf was constructed and secretion of rhFGF21 protein was achieved in this strain. Eleven signal peptides (SP_*phoD*_, SP_*pel*_, SP_*ywbN*_, SP_*lipA*_, SP_*protA*_, SP_*ywmC*_, SP_*dacB*_, SP_*nprE*_, SP_*yddT*_, SP_*yoqM*_, SP_*yvcE*_) were screened and explored, and the results indicate that SP_*dacB*_ is the most suitable for the production of rhFGF21 in *B. subtilis*. The LC–MS and ERK1/2 phosphorylation bioactivity assay also proved the secreted rhFGF21 from *B. subtilis* was well processed by signal peptidase and had a comparable potency with the functional rhFGF21 standard sample. The work presented herein illustrates a comprehensive method to enhance the production of a heterologous protein in *B. subtilis.*

## Methods

### Bacterial strains, plasmids and growth conditions

In this study, bacterial strains and plasmids used are listed in Tables 1 and 2. *E. coli* DH5α was used as a host for cloning and plasmid preparation, and was incubated in Luria–Bertani (LB) liquid medium (1% (w/v) tryptone, 0.5% (w/v) yeast extract and 1% (w/v) NaCl) or on LB agar plates. *B. subtilis* 1A751 was used as a host for the expression of rhFGF21. Unless otherwise specified, integrated *B. subtilis* mutants were selected on LB agar plates, and cultivated in SR medium (1.5% (w/v) tryptone, 2.5% (w/v) yeast extract and 0.3% (w/v) K_2_HPO_4_, pH 7.2) at 37 °C. The plasmid pMATE, an *E. coli/B. subtilis* shuttle vector which is derived from pMA5 and replaces a constitutive promoter P_*hpaII*_ with a maltose-inducible promoter P_*malA*_ [27], was used as the expression protein vector. All vectors for rhFGF21 expression in *B. subtilis* are pMATE derivatives. The plasmid pDF is an integration vector which was used for overexpressing chaperones and transport proteins, it was derived from pDL by both the front and back parts of *amyE*. *E. coli* and *B. subtilis* transformants were selected on LB agar plates and supplemented with ampicillin (100 μg/mL), chloramphenicol (5 μg/mL) or kanamycin (20 μg/mL) depending on the plasmid antibiotic marker. All strains were incubated while shaking at 200 rpm. When required, 0.2% (w/v) maltose was added to the medium immediately after inoculation.

### General molecular biology techniques

Standard molecular techniques including *E. coli* transformation were carried out according to Sambrook et al. [55]. *B. subtilis* was naturally transformed using the “Paris Method” [56, 57]. PCR was performed using Prime STAR Max DNA Polymerase (TaKaRa, Japan). DNA fragments and PCR products were excised from 0.8% agarose gel and purified by E.Z.N.A.^Tm^ Gel Extraction Kit (200) (Omega Bio-tek, Inc., USA) according to the manufacturer’s instructions. Construction of recombinant plasmids was performed using the ClonExpress ^Tm^ II One Step Cloning Kit (Vazyme Biotech Co. Ltd, China) according to the manufacturer’s instructions. E.Z.N.A.^Tm^ Plasmid Mini Kit I (Omega Bio-tek, Inc., USA) was used for plasmid extraction according to the manufacturer’s instructions. Genomic DNA isolation was carried out using the TIANamp Bacteria DNA Kit (Tiangen Biotech (Beijing) Co., Ltd., China). All DNA constructs were sequenced by GENEWIZ (Suzhou, China).

### Markerless deletion of extracellular proteases

All primers used for markerless gene deletion were synthesized by GENEWIZ and are listed in Additional file 5: Table S1. In this study, the six genes-deficient (*bpr*, *epr*, *wprA*, *mpr*, *nprB* and *vpr*) strain *B. subtilis* Kno6 was obtained using marker-free gene deletion [58, 59]. The 0.9-kb *cat* (C) fragment, which contains the entire coding domain of the *cat* gene, was amplified from the plasmid pDL using the primer pair Cm-F/Cm-R. The 1.2-kb *araR* (R) fragment was amplified from the *B. subtilis* 168 genome with the primers araR-F and araR-R. The upstream and downstream domains of genes (*bpr* and *epr* and *wprA* and *mpr* and *nprB* and *vpr*) (G) were amplified from the *B. subtilis* 168 genome DNA using the primer pairs UP-F/UP-R and DN-F/DN-R, respectively. The method of splicing by overlapped extension PCR (SOE-PCR) [60] used the primers UP-F and DN-R to achieve the fusion fragment UP-DN-C-R-G. Eventually, the fusion fragment was naturally transformed into *B. subtilis* 1A751 using the “Paris Method” [57], yielding the mutant strain Kno6. Markerless deletion of the six extracellular proteases in 1A751 was then validated by DNA sequencing.

### Construction of the expression vectors containing different mini-cistron cassettes and signal peptides

The recombinant plasmids were generated using ClonExpress^Tm^ II One Step Cloning Kit (Vazyme Biotech Co., Ltd., Nanjing, China) according to the instructions given by the manufacturer. All cloning techniques and transformation of *E. coli*/*B. subtilis* were performed as previously described [16, 18]. In this study, the nucleic acid sequence of rhFGF21 has been codon optimized to permit expression in *B. subtilis* and was obtained by Total Gene Synthesis (GENEWIZ). The pMATEF plasmid, which was used for rhFGF21 inducible expression, was generated by ligation of a signal peptide sequence SP_*pelB*_ amplified by PCR using the *B. subtilis* 168 genome as the template and the primers SP_*pelB*_-F/SP_*pelB*_-R (Additional file 6: Table S2), an insertion fragment rhFGF21 obtained by PCR using primers rhFGF21-F/rhFGF21-R, and the linear vector backbone of the pMATE plasmid amplified with primers pMATE-F/pMATE-R. The pMA5F plasmid was generated by ligation of an insertion fragment rhFGF21 with SP_*pelB*_ obtained by PCR using primers rhFGF21-F2/rhFGF21-R2 and the linear vector backbone of pMA5 amplified with primers pMA5-F/pMA5-R by ClonExpress^Tm^ II One Step Cloning Kit method.

The assembly of pMATEFc1 is used as an example below to illustrate the procedure for the construction of the mini-cistron cassette vector. The cistron1 fragment was generated by hybridization of equimolar amounts of complementary oligonucleotide in 1× STE buffer (10 mM Tris, 100 mM NaCl, and 1 mM EDTA), heated to 95 °C for 5 min, followed by incubation at room temperature for 1 h [61]. The pMATEF vector backbone was amplified from the pMATEF plasmid, using the primers pMATEF-F/pMATEF-R. Then, the pMATEFc1 plasmid containing these two parts was generated using ClonExpress^Tm^ II One Step Cloning Kit according to the instructions of the manufacturer and was directly transformed into *E. coli* DH5α. The resulting plasmid pMATEFc1 was identified and validated via colony PCR and sequencing (GENEWIZ). Then, pMATEFc2, pMATEFc3, pMATEFc4, pMATEFc5, pMATEFc6 and pMATEFc7 were constructed in a similar manner.

To investigate the influence of signal peptides on the expression of rhFGF21 in *B. subtilis*, 12 signal peptides (SP_*phoD*_, SP_*pel*_, SP_*ywbN*_, SP_*lipA*_, SP_*protA*_, SP_*ywmC*_, SP_*dacB*_, SP_*nprE*_, SP_*yddT*_, SP_*yoqm*_ *and* SP_*yvce*_) were compared. SP_*phoD*_, Sp_*pel*_, SP_*ywbN*_, SP_*lipA*_, SP_*ywmC*_, SP_*dacB*_, SP_*nprE*_, SP_*yddT*_, SP_*yoqm*_ *and* SP_*yvce*_ were amplified using *B. subtilis* 168 genome as the template (primers are listed in Additional file 6: Table S2). The SP_*protA*_ signal peptide was amplified by PCR using Total Gene Synthesis (GENEWIZ) using the primers SP_*protA*_-F/SP_*protA*_-R. The vector backbone was amplified from pMATEFc5 with primers pMATEFc5-F/pMATEFc5-R. Using the ClonExpress^Tm^ II One Step Cloning Kit method as described above, the different signal peptides vectors were constructed.

### Construction of the integration vectors

The plasmid pDF, which was used as an integration vector for 11 chaperone genes (*blsecA*, *csaA*, *dnak*, *ffh*, *ftsY*, *groESL*, *prsA*, *qssecA*, *scr*, *SRP* and *yrdF*) was generated by ligation of an insertion fragment P_*grac*_ obtained by PCR from pHT43 using primers P_*grac*_-F/P_*grac*_-R and the linear vector backbone of pDL amplified with primers pDL-F/pDL-R. Sixteen genes (*csaA*, *dnak*, *ffh*, *ftsY*, *prsA*, *qssecA*, *scr*, *yrdF*) and one gene operon *groESL* were amplified using relevant primers (Additional file 6: Table S2) and *B. subtilis* 168 genomic DNA as the template. The gene *qssecA* was truncated [62] and amplified from *B. subtilis* 168 genomic DNA as the template, using primers qssecA-F/qssecA-R. The gene *blsecA* was amplified from *B. licheniformis* ATCC 14580, using primers blsecA-F/blsecA-R. The *SRP* operon contains the gene *ffh*, *hbs*, *scr*, and its own RBS were amplified from *B. subtilis* 168 genomic DNA using the primers ffh-F/ffh-R, hbs-F/hbs-R and scr-F/scr-R. The SOE-PCR method used the primers ffh-F/scr-R to make the fusion fragment of the *SRP* operon. The vector backbone was amplified from pDF with primers pDF-F/pDF-R. Using ClonExpress^Tm^ II One Step Cloning Kit method as described above, all of the integration plasmids were constructed successfully.

### SDS-PAGE analysis

One mL of culture sample was collected when fermentation was completed and the sample was centrifuged (12,000*g*, 10 min, 4 °C). The supernatant fraction was collected and the pellet was re-suspended in lysis buffer (50 mM Tris–HCl, pH 8, 2.5 mM EDTA). The two fractions were treated with 5× SDS-PAGE sample buffer, then boiled for 20 min and proteins were separated via SDS-PAGE using the NuPAGE 12% Bis–Tris Gel (Novex by Life Technologies, USA) in combination with MOPS SDS Running Buffer (Invitrogen Life Technologies, USA). Page Ruler Prestained Protein Ladder (Invitrogen Life Technologies, USA) was used to determine the apparent molecular weight of separated proteins. Proteins were visualized with Coomassie Brilliant Blue.

### Fermentation experiments

All of the recombinant strains were activated by LB agar plates and LB liquid medium. After that, the recombinant strains were inoculated into 250 mL Erlenmeyer flasks containing SR medium for 24 h at 37 °C, shaking at 220 rpm and in the presence of 0.2% (w/v) maltose to induce the expression of rhFGF21.

### Western blot

Samples were analyzed by SDS-PAGE for immunoblotting. Proteins were separated on 12% Bis–Tris Gel and transferred to nitrocellulose membranes. The blots were first probed with mouse primary antibodies and then with the anti-mouse IgG (H + L) secondary antibody. Protein GroEL was using as a reference. Detection was performed using CD/DAB substrate kit (Thermo Scientific, USA).

### LC–MS analysis

FGF21 protein was purified with Ni–NTA based affinity chromatography for LC–MS analysis. Before LC–MS analysis, the purified secreted rhFGF21 with C-terminal his-tag was analyzed by SDS-PAGE using rhFGF21 standard sample provided by Novo nordisk as a positive control. The sample was filtered using 0.22 µm filters and then directly injected for analysis. LC–MS analysis was carried out using an ACQUITY UPLC BEH shield RP18 equipped with a column (1.7 µm, 2.1 × 150 mm). The mobile phase was 0.1% TFA in H_2_O (solvent A) and 0.07% TFA in ACN (solvent B). The samples were analyzed at 60 °C and 1.0 mL/min by UV absorbance at 215 nm.

### ERK1/2 phosphorylation bioactivity assay

HEK-hBKL cells with stable over-expression of βKlotho (Klb) were treated with hFGF21 sample and purified rhFGF21 from *B. subtilis* as indicated for 5 min and subsequently lysed. Total ERK phosphorylation was assessed using an AlphaScreen SureFire Phospho ERK1/2 Assay Kit (Perkin Elmer, Waltham, MA) according to the manufacturer’s instructions and an EnVision Multilabel Microplate Reader Model 2103 (Perkin Elmer) with the AlphaScreen HTS Turbo option was used for signal detection. The rhFGF21 standard sample provided by Novo nordisk was used as a positive control for ERK1/2 Assay.

## Additional files

**Additional file 1: Figure S1.** The relative expression level of rhFGF21 from different *B. subtilis* strains.

**Additional file 2: Figure S2.** SDS-PAGE analysis of the purified secreted rhFGF21 with C-terminal His-tag *from B. subtilis*. Lane 1 represents the protein marker. Lane 2–4 represents different load volume of purified rhFGF21 from *B. subtilis.* Lane 5 (std) represents the rhFGF21 standard sample as a control. Arrow indicates the band of rhFGF21 protein.

**Additional file 3: Figure S3.** LC–MS analysis of rhFGF21 protein expressed and purified from *B. subtilis* Kno6cf. (A). HPLC analysis result of rhFGF21 from Kno6cf, the red indicator represents the intact secreted mature rhFGF21. (B) MS analysis result of rhFGF21 from Kno6cf.

**Additional file 4: Figure S4.** ERK1/2 phosphorylation assay of hFGF21 standard and rhFGF21 from Kno6cf in HEK-hBKL cells with stable over-expression of βKlotho (Klb).

**Additional file 5: Table S1.** Summary of in vitro activities of rhFGF21 from Kno6cf and hFGF21 standard sample.

**Additional file 6: Table S2.** All primers used in this study.
