# Supplementary material for: High-efficiency expression and secretion of human FGF21 in Bacillus subtilis by intercalation of a mini-cistron cassette and combinatorial optimization of cell regulatory components
Source: Microb Cell Fact. 2019 Jan 28;18:17. doi: 10.1186/s12934-019-1066-4 (PMC6348689; doi:10.1186/s12934-019-1066-4)

**High-Efficiency expression and secretion of human FGF21 in *Bacillus subtilis* by intercalation of a mini-cistron cassette and combinatorial optimization of cell regulatory components**

Dandan Li^1,2,#^, Gang Fu^2,3,#^, Ran Tu^2^, Zhaoxia Jin^1*^ and Dawei Zhang^2,3*^

^1^School of Biological Engineering, Dalian Polytechnic University, Dalian 116034, People’s Republic of China.

^2^Tianjin Institute of Industrial Biotechnology, Chinese Academy of Sciences, Tianjin 300308, People’s Republic of China.

^3^Key Laboratory of Systems Microbial Biotechnology, Chinese Academy of Sciences, Tianjin 300308, People’s Republic of China.

^#^ DL and GF are equally contributed to this work.

* Corresponding author: Zhaoxia Jin, E-mail address: [jinzx2018@163.com](mailto:jinzx2018@163.com);

Dawei Zhang, E-mail address: zhang_dw@tib.cas.cn.

**Additional file 2: Figure S2.** SDS-PAGE analysis of the purified secreted rhFGF21 with C-terminal His-tag *from B. subtilis*. Lane 1 represents the protein marker. Lane 2-4 represents different load volume of purified rhFGF21 from *B. subtilis.* Lane 5 (std) represents the rhFGF21 standard sample as a control. Arrow indicates the band of rhFGF21 protein.


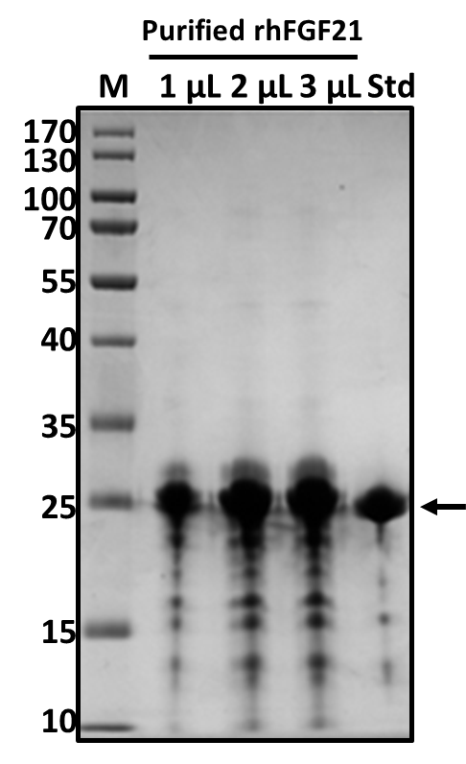

Supplement: Supplementary file 2 — Additional file 2: Figure S2. SDS-PAGE analysis of the purified secreted rhFGF21 with C-terminal His-tag from B. subtilis. Lane 1 represents the protein marker. Lane 2–4 represents different load volume of purified rhFGF21 from B. subtilis. Lane 5 (std) represents the rhFGF21 standard sample as a control. Arrow indicates the band of rhFGF21 protein. [file 12934_2019_1066_MOESM2_ESM.docx]
