# Supplementary material for: High-efficiency expression and secretion of human FGF21 in Bacillus subtilis by intercalation of a mini-cistron cassette and combinatorial optimization of cell regulatory components
Source: Microb Cell Fact. 2019 Jan 28;18:17. doi: 10.1186/s12934-019-1066-4 (PMC6348689; doi:10.1186/s12934-019-1066-4)

**High-Efficiency expression and secretion of human FGF21 in *Bacillus subtilis* by intercalation of a mini-cistron cassette and combinatorial optimization of cell regulatory components**

Dandan Li^1,2,#^, Gang Fu^2,3,#^, Ran Tu^2^, Zhaoxia Jin^1*^ and Dawei Zhang^2,3*^

^1^School of Biological Engineering, Dalian Polytechnic University, Dalian 116034, People’s Republic of China.

^2^Tianjin Institute of Industrial Biotechnology, Chinese Academy of Sciences, Tianjin 300308, People’s Republic of China.

^3^Key Laboratory of Systems Microbial Biotechnology, Chinese Academy of Sciences, Tianjin 300308, People’s Republic of China.

^#^ DL and GF are equally contributed to this work.

* Corresponding author: Zhaoxia Jin, E-mail address: [jinzx2018@163.com](mailto:jinzx2018@163.com);

Dawei Zhang, E-mail address: zhang_dw@tib.cas.cn.

**Additional file 3: Figure S3.** LC-MS analysis of rhFGF21 protein expressed and purified from B. subtilis Kno6cf. (A). HPLC analysis result of rhFGF21 from Kno6cf, the red indicator represents the intact secreted mature rhFGF21. (B) MS analysis result of rhFGF21 from Kno6cf.


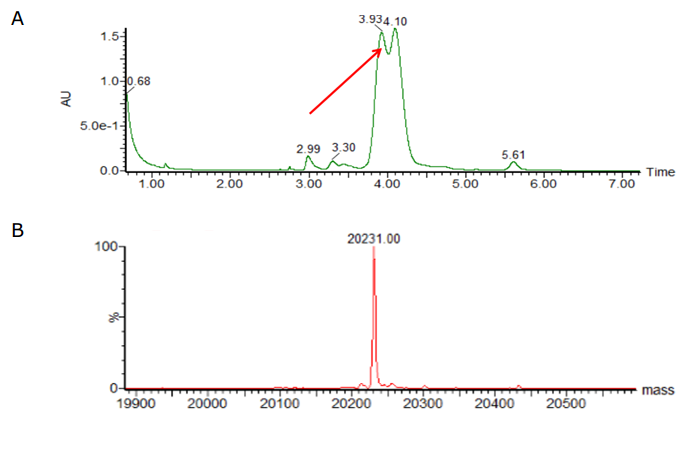

Supplement: Supplementary file 3 — Additional file 3: Figure S3. LC–MS analysis of rhFGF21 protein expressed and purified from B. subtilis Kno6cf. (A). HPLC analysis result of rhFGF21 from Kno6cf, the red indicator represents the intact secreted mature rhFGF21. (B) MS analysis result of rhFGF21 from Kno6cf. [file 12934_2019_1066_MOESM3_ESM.docx]
