# Supplementary material for: High-efficiency expression and secretion of human FGF21 in Bacillus subtilis by intercalation of a mini-cistron cassette and combinatorial optimization of cell regulatory components
Source: Microb Cell Fact. 2019 Jan 28;18:17. doi: 10.1186/s12934-019-1066-4 (PMC6348689; doi:10.1186/s12934-019-1066-4)
Supplement: Supplementary file 5 — Additional file 5: Table S1. Summary of in vitro activities of rhFGF21 from Kno6cf and hFGF21 standard sample. [file 12934_2019_1066_MOESM5_ESM.docx]

**High-Efficiency expression and secretion of human FGF21 in *Bacillus subtilis* by intercalation of a mini-cistron cassette and combinatorial optimization of cell regulatory components**

Dandan Li^1,2,#^, Gang Fu^2,3,#^, Ran Tu^2^, Zhaoxia Jin^1*^ and Dawei Zhang^2,3*^

^1^School of Biological Engineering, Dalian Polytechnic University, Dalian 116034, People’s Republic of China.

^2^Tianjin Institute of Industrial Biotechnology, Chinese Academy of Sciences, Tianjin 300308, People’s Republic of China.

^3^Key Laboratory of Systems Microbial Biotechnology, Chinese Academy of Sciences, Tianjin 300308, People’s Republic of China.

^#^ DL and GF are equally contributed to this work.

* Corresponding author: Zhaoxia Jin, E-mail address: [jinzx2018@163.com](mailto:jinzx2018@163.com);

Dawei Zhang, E-mail address: zhang_dw@tib.cas.cn.

**Additional file 5: Table S1.** Summary of *in vitro* activities of rhFGF21 from Kno6cf and hFGF21 standard sample.

| **ERK1/2 Phospho activity** | **rhFGF21 from Kno6cf** | **hFGF21** |
| --- | --- | --- |
| **EC50 (nM)** | 1.449 | 2.866 |
| **Top** | 34598 | 26427 |
| **Bottom** | 407.7 | 531.8 |
