# Supplementary material for: High-efficiency expression and secretion of human FGF21 in Bacillus subtilis by intercalation of a mini-cistron cassette and combinatorial optimization of cell regulatory components
Source: Microb Cell Fact. 2019 Jan 28;18:17. doi: 10.1186/s12934-019-1066-4 (PMC6348689; doi:10.1186/s12934-019-1066-4)
Supplement: Supplementary file 6 — Additional file 6: Table S2. All primers used in this study. [file 12934_2019_1066_MOESM6_ESM.docx]

**High-Efficiency expression and secretion of human FGF21 in *Bacillus subtilis* by intercalation of a mini-cistron cassette and combinatorial optimization of cell regulatory components**

Dandan Li^1,2,#^, Gang Fu^2,3,#^, Ran Tu^2^, Zhaoxia Jin^1*^ and Dawei Zhang^2,3*^

^1^School of Biological Engineering, Dalian Polytechnic University, Dalian 116034, People’s Republic of China.

^2^Tianjin Institute of Industrial Biotechnology, Chinese Academy of Sciences, Tianjin 300308, People’s Republic of China.

^3^Key Laboratory of Systems Microbial Biotechnology, Chinese Academy of Sciences, Tianjin 300308, People’s Republic of China.

^#^ DL and GF are equally contributed to this work.

* Corresponding author: Zhaoxia Jin, E-mail address: [jinzx2018@163.com](mailto:jinzx2018@163.com);

Dawei Zhang, E-mail address: zhang_dw@tib.cas.cn.

**Additional file 6: Table S2.** All primers used in this study.

| Primers | Sequence (5’-3’) |  |  |  |
| --- | --- | --- | --- | --- |
| rhFGF21.F | catccgattccggatagcag |  |  |  |
| rhFGF21.R | ttaatgatgatgatgatgatggctcg |  |  |  |
| rhFGF21.F2 | taaTAAGGAGGatttacatatgaaatacctattgcctacggcagc |  |  |  |
| rhFGF21.R2 | gctatgcgagccatcatcatcatcatcattaa |  |  |  |
| pMATE-vector.F | agaagtctcgttccgacagttgg |  |  |  |
| pMATE-vector.R | aattattccccctagctaattttcgtTTAATTATAAATTAAG |  |  |  |
| pMA5-vector.F | catatgagttatgcagtttgtagaatgc |  |  |  |
| pMA5-vector.R | gtgtgctctgcgaggctg |  |  |  |
| pMATEF-vector.F | catccgattccggatagcagcc |  |  |  |
| pMATEF-vector.R | TTTAACTTAATTTATAATTAAacgaaaattagctagggggaataatt |  |  |  |
| p*_malA_*.F | cgcGGATCCCTTTTGTCCCCTGCCTTTTCT |  |  |  |
| p*_malA_*.R | cggGGTACCATGACGACCTCCTTGATAAatTTTACAATTCCATTTA |  |  |  |
| CIS1.F | TAAacgatgaaatacctattgcctacggcagc |  |  |  |
| CIS1.R | ttcttctcctttacTcATaattattccccctagctaattttcgtTT |  |  |  |
| CIS2.F | cttgtatcatgaaatacctattgcctacggcagc |  |  |  |
| CIS2.R | GTTTTTGGCGTCTTCCATaattattccccctagctaattttcgtTT |  |  |  |
| CIS3.F | atctgttcaatgaaatacctattgcctacggcagc |  |  |  |
| CIS3.R | GAATGATTTTTTCTTCATaattattccccctagctaattttcgtTT |  |  |  |
| CIS4.F | aggttttatatgaaatacctattgcctacggcagc |  |  |  |
| CIS4.R | ataaataccatatttcataattattccccctagctaattttcgtTT |  |  |  |
| CIS5.F | aTAAGGAGGatttacatatgaaatacctattgcctacggcagc |  |  |  |
| CIS5.R | TTTGTTATTGTCTGCCATaattattccccctagctaattttcgtTT |  |  |  |
| CIS6.F | ataaggaggaataaatgaaatacctattgcctacggcagc |  |  |  |
| CIS6.R | ttaatcgatacataattattccccctagctaattttcgtTT |  |  |  |
| CIS7.F | ggatgattgaatgaaatacctattgcctacggcagc |  |  |  |
| CIS7.R | tccttgaacataattattccccctagctaattttcgtTT |  |  |  |
| SP*_pelB_*.F | atgaaatacctattgcctacggcagc |  |  |  |
| SP*_pelB_*.R | gctcaacccgcaatggcc |  |  |  |
| SP*_phoD_*.F | atggcatacgacagtcgttttgatgaa |  |  |  |
| SP*_phoD_*.R | ggttggggcctttgaagtaaatgct |  |  |  |
| SP*_pel_*.F | ATGAAAAAAGTGATGTTAGCTACGGCTTT |  |  |  |
| SP*_pel_*.R | GCTGGCGCGAACGCA |  |  |  |
| SP*_ywbN_*.F | atgagcgatgaacagaaaaagccag |  |  |  |
| SP*_ywbN_*.R | gggcagccgttgcg |  |  |  |
| SP*_lipA_*.F | ATGAAATTTGTAAAAAGAAGGATCATTGCACTTGT |  |  |  |
| SP*_lipA_*.R | CGTCAGCAAAAGCCGCT |  |  |  |
| SP*_protA_*.F | ATGAAAAAGAAAAACATTTATTCAATTCGTAAACTAGGTGT |  |  |  |
| SP*_protA_*.R | CGTAACACCTGCTGCAAATGCT |  |  |  |
| SP*_ywmC_*.F | atgaagaaaagattttcactgatcatgatgacagg |  |  |  |
| SP*_ywmC_*.R | ctttttggattaacttcacctgcttttgca |  |  |  |
| SP*_dacB_*.F | ATGCGCATTTTCAAAAAAGCAGTATTCG |  |  |  |
| SP*_dacB_*.R | ACCGTAAATGTGAATACAGCACATGCT |  |  |  |
| SP*_nprE_*.F | ATGGGTTTAGGTAAGAAATTGTCTGTTGCT |  |  |  |
| SP*_nprE_*.R | GCCTGCCAGGTGTTCAGGCT |  |  |  |
| SP*_yddT_*.F | ATGAGAAAGAAAAGAGTTATTACTTGTGTTATGGCT |  |  |  |
| SP*_yddT_*.R | TTTACCTGCAGGTTACGCTTCTGCA |  |  |  |
| SP*_yoqm_*.F | ATGAAATTAAGAAAAGTATTGACTGGTTCTGTT |  |  |  |
| SP *_yoqm_*.R | TTCTGCTTCTCCTGCATTCGCT |  |  |  |
| SP*_yvce_*.F | ATGAGAAAGAGTTTAATTACACTTGGTTTGGC |  |  |  |
| SP*_yvce_*.R | CATTTACAAGTAAAACTGCATCGGCG |  |  |  |
| BlsecA.F | atgcttggaattttaaataaagtgtttgat |  |  |  |
| BlsecA.R | ttattctgttcttccgcagcagtttttata |  |  |  |
| CsaA.F | TTATCCGATTTTTGTGCCGTTTGGG |  |  |  |
| CsaA.R | ATGGCAGTTATTGATGACTTTGAGAA |  |  |  |
| DnaK.F | TTATTTTTTGTTTTGGTCGTCGTTTACTTCTT |  |  |  |
| DnaK.R | GTGAGTAAAGTTATCGGAATCGACTTAGGAAC |  |  |  |
| Ffh.F | ATGGCATTTGAAGGATTAGCCGAC |  |  |  |
| Ffh.R | TTACATAAAAGGTAGCTTAAACCCTTTTTTCTT |  |  |  |
| Ftsy.F | ATGAGCTTTTTTAAAAAATTAAAAGAGAAAATCACAAAAC |  |  |  |
| Ftsy.R | TTAATCGTCGGCTTTTTCCACTAAATCTGAAA |  |  |  |
| GroESL.F | TTGTTAAAGCCATTAGGTGATCGCG |  |  |  |
| GroESL.R | TTACATCATTCCACCCATACCGCCC |  |  |  |
| PrsA.F | TTATTTAGAATTGCTTGAAGATGAAGAAGTGCTG |  |  |  |
| PrsA.R | ATGAAGAAAATCGCAATAGCAGC |  |  |  |
| QssecA.F | TCAAACATTTTATTTAAAATTCCAAGCAT |  |  |  |
| QssceA.R | CCGCTTCGTGAGTATCAAATGGAATAG |  |  |  |
| Scr.F | CGTTTGGGTCCTGCGCAAT |  |  |  |
| Scr.R | AGGGTTGCCTGGGCCGAGC |  |  |  |
| YrdF.F | CTACTCTATTATTATTTTAAATTCGTCCTTTAGTTCTTCT |  |  |  |
| YrdF.R | ATGAGAAAAATAATAATAGATGGAAGAGACTTTGAAAAT |  |  |  |
| Hbs.f | TTATTTTCCGGCAACTGCGTCTT |  |  |  |
| Hbs.r | ATGAACAAAACAGAACTTATCAATGCGGTT |  |  |  |
| Pgrac.F | AAAGGAGGTAAGGATCACTAGAAAATTTTTTAAA |  |  |  |
| Pgrac.F | TTCCTCCTTTaattggtgttggttgttgt |  |  |  |
| PDF.F | TAATAACCGGGCAGGCCATGTCT |  |  |  |
| PDF.R | CCAGTGCAGGAGCTCTTAGCATATTATG |  |  |  |
| UP.F | AATCTCGGCAATGAAAGAAGCGG |  |  |  |
| UP.R | TTTCATCCCCCTTTTTCAACATGCT |  |  |  |
| DN.F | GTGGAAAAAAAGCTGCCGTCATT |  |  |  |
| DN.R | CCGACAGCTTTGTTGTGCTGATA |  |  |  |
| Cm.F | TCTTCAACTAAAGCACCCATTAGTTCAACAAA |  |  |  |
| Cm.R | TTATTCATTCAGTTTTCGTGCGGACTGG |  |  |  |
| araR.F | ATGTTTTCTTACAAAGAACGCTGTGATAT |  |  |  |
| araR.R | TTATTCATTCAGTTTTCGTGCGGACT |  |  |  |
| G1.F | ATGAGGAAAAAAACGAAAAACAGACTCATCA |  |  |  |
| G1.R | TTAATTTTCTGTGTTCATATTAAGTTTTCCATTCGC |  |  |  |
| G2.F | ATGAAAAACATGTCTTGCAAACTTGT |  |  |  |
| G2.R | TTACTTTTTCGGTTTGACGGCGTTCAG |  |  |  |
| G3.F | ATGAAACGCAGAAAATTCAGCTCGG |  |  |  |
| G3.R | TTACTTTTCAACAACAACTTTTGCTTCCT |  |  |  |
| G4.F | ATGAAATTAGTTCCAAGATTCAGAAAACAATGGT |  |  |  |
| G4.R | TTAGACGGAGTCTTTTTTGCTTTTGCC |  |  |  |
| G5.F | TTGCGCAACTTGACCAAGAC |  |  |  |
| G5.R | TCATAGAATGCCGACAGCCTCATACG |  |  |  |
| G6.F | TTGAAAAAGGGGATCATTCGCTTTCTG |  |  |  |
| G6.R | CTTATTCAACAGTGAAAGGTTCTTCGGTCAAA |  |  |  |
| UP2.F | CTTGGAAGGCGAGACGGTTGG |  |  |  |
| UP2.R | GATTCATCTCCTTTTTCTATGATGTTTGATATATATATCG |  |  |  |
| DN2.F | CCAAAAACCTTTAAGATTTGCATTCCA |  |  |  |
| DN2.R | TAGCCCATCATTGGGATATGAAGCCC |  |  |  |
| UP3.F | AAAAAGGCATAAGCAGTTTTTTATCGG |  |  |  |
| UP3.R | GTTATCCCTCCTGCAAAATAATGAATCT |  |  |  |
| DN3.F  DN3.R | CCAAAAAGCGGTGCTCGATGC  TATAAAGCTCGCCATTGTAAATAATGGTGTACGTAT |  |  |  |
| UP4.F | TTGCGACGGATGAATTGCTGAAGCT |  |  |  |
| UP4.R | TTTGTCATCTCCCTCCTTTAGTGTCA |  |  |  |
| DN4.F | TAAGACGGAGTCTTTTTTTATTTCGTTTTTAAGAA |  |  |  |
| DN4.R | GAGGAAAAAGATGAAAAAGCATATCAGCATGC |  |  |  |
| UP5.F | TCATTGACCGATACATGGAGGAACTAAATGCA |  |  |  |
| UP5.R | AACACCACATCCTTCCTATTTTGGAAT |  |  |  |
| DN5.F | GCAAACAAAAACAGTCAGGACACAGAG |  |  |  |
| DN5.R | ATTTTGGCAAATTAATATGATATAGTTGGCTTAACT |  |  |  |
| UP6.F | TCCACTTTATTCATAGGGATGAGATCATTTTTCTT |  |  |  |
| UP6.R | TGTGTTTCCCCCTTTGTATTTAGAAAAAATGTGA |  |  |  |
| DN6.F | AAAAAGCCCTGCCGATTCGG |  |  |  |
| DN6.R | CAGAAACGGCCTTACAGCAAACC |  |  |  |
